# Neurobiomechanical mechanism of Tai Chi to improve upper limb coordination function in post-stroke patients: a study protocol for a randomized controlled trial

**DOI:** 10.1186/s13063-023-07743-w

**Published:** 2023-12-04

**Authors:** Qiurong Xie, Jinsong Wu, Qi Zhang, Yanxin Zhang, Bo Sheng, Xiaoling Wang, Jia Huang

**Affiliations:** 1 Fujian University of Traditional Chinese Medicine, Fuzhou, 350122 China; 2 Key Laboratory of Orthopedics & Traumatology of Traditional Chinese Medicine and Rehabilitation (Fujian University of TCM), Ministry of Education, Fuzhou, 350122 China; 3 The University of Auckland, Auckland, New Zealand 1142; 4 Shanghai University, Shanghai, 200444 China

**Keywords:** Tai Chi, Stroke, Upper limb function, Corticomuscular coupling, Brain imaging, Randomized controlled trial

## Abstract

**Background:**

Upper limb dysfunction seriously affects the ability of stroke patients to perform activities of daily living. As a popular exercise therapy, Tai Chi may become an alternative intervention. However, the neurophysiological mechanism by which Tai Chi improves upper limb dysfunction in stroke patients is still unclear, which limits its further promotion and application. Therefore, conducting a strict randomized clinical trial is necessary to observe how Tai Chi affects upper limb dysfunction in stroke patients and to explore its neurophysiological mechanism.

**Methods/design:**

This report describes a randomized, parallel-controlled trial with distributive concealment and evaluator blinding. A total of 84 eligible participants will be randomly assigned to the Tai Chi group or the control group in a 1:1 ratio. The participants in the Tai Chi group will receive 4 weeks of Tai Chi training: five 60-min sessions a week for a total of 20 sessions. The participants in the control group will not receive Tai Chi training. Both groups will receive medical treatment and routine rehabilitation training. The primary outcome measure is the mean change in the Fugl-Meyer Assessment Upper Extremity (FMA-UE) scale score between baseline and 4 weeks; the secondary outcomes are the mean changes in kinematic characteristics and the Wolf Motor Function Test (WMFT) and Stroke Impact Scale (SIS) scores. In addition, the corticomuscular coupling level and near-infrared brain functional imaging will be monitored to explore the mechanism by which Tai Chi improves upper limb function of stroke patients.

**Discussion:**

This randomized controlled trial will examine the effectiveness of Tai Chi in stroke patients with upper limb dysfunction and explore the neurophysiological mechanism. Positive results will verify that Tai Chi can improve upper limb function of stroke patients.

**Trial registration:**

Chinese Clinical Trial Registration Center, ChiCTR2200061376 (retrospectively registered). Registered June 22, 2022. http://www.chictr.org.cn/listbycreater.aspx. Manuscript Version: 3.0 Manuscript Date: October 10, 2023.

**Supplementary Information:**

The online version contains supplementary material available at 10.1186/s13063-023-07743-w.

## Background

Upper limb motor dysfunction after stroke is a significant problem that affects the quality of life of stroke patients [1]. Research shows that about 85% of stroke patients have upper limb dysfunction when they first get sick. Approximately 15% of these patients recover hand function to about 50% of their original level, while only 3% of patients recover hand function that is > 70% of their original level [2]. Upper limb function is of great significance to individuals and society because the upper limb motor ability is necessary for activities of daily living and affects quality of life [3].

Studies have confirmed that Tai Chi has a positive clinical effect on upper limb dysfunction in stroke patients. A meta-analysis showed that upper limb rehabilitation training is one of the most widely used and effective measures for patients with upper limb dysfunction after stroke. Some scholars [4] have evaluated the efficacy of Tai Chi in treating post-stroke upper limb dysfunction and have systematically reviewed the relevant randomized controlled trials. The results support the conclusion that Tai Chi combined with upper limb rehabilitation training is significantly better than upper limb rehabilitation training alone to treat post-stroke upper limb dysfunction. Indeed, Tai Chi combined with rehabilitation training significantly improves upper limb function compared with control treatment [5].

Coupling of the sensorimotor frontal-parietal network and muscle activation patterns is an essential mechanism for functional recovery of upper limbs. Bilateral coupling between the ascending and descending cortex and muscle in the sensorimotor control circuit is weakened in stroke patients, resulting in abnormal upper limb muscle coordination and movement patterns as well as compensatory movement [6–8]. When the elbow of a stroke patient moves, β and γ functional corticomuscular coupling (FCMC) of the anterior deltoid and brachial muscles change. A decrease in FCMC in the biceps brachii and an increase in FCMC in the deltoid muscle are related to a reduction in elbow flexion and excessive abduction of the shoulder joint [9]. Therefore, abnormal movement patterns in stroke patients, including slow movement, poor fluency, and greater movement variability, lead to compensatory trunk movement [10, 11].

Researchers have also found that enhanced coupling of the sensorimotor frontal-parietal cortex and muscle activation patterns is closely related to upper limb function recovery. Participants with high resting-state functional connectivity (rsFC) within and between motor networks—the contralateral primary motor cortex (M1) and the ipsilateral supplementary motor area SMA)—and frontal-parietal networks—between the dorsolateral prefrontal cortex (DLPFC) and the medial ventrolateral prefrontal cortex—have better hand function [12]. The functional connectivity (FC) of the ipsilateral premotor cortex (PMC) and the frontal-parietal region (bilateral precuneus and the DLPFC) are positively correlated with motor function. Compared with the healthy side, the FC of the ipsilateral somato-motor/sensory network (SMN) increases with bilateral frontal lobe (DLPFC) and the ipsilateral posterior parietal cortex (Pcu) during hand movement. The FC of the ipsilateral PMC and frontal-parietal region (bilateral Pcu and contralateral DLPFC) is positively correlated with motor function [13]. The motor frontal-parietal connection may be related to the neural processes related to motor execution and may be a potential biomarker for motor recovery after stroke [14].

Coupling of the sensorimotor frontal-parietal cortex and the muscle may be an essential mechanism by which Tai Chi improves upper limb function. Studies have confirmed that Tai Chi activates sensorimotor function, the frontal-parietal lobe, and other brain regions in healthy individuals. Tai Chi improves the functional integration of motor and sensory areas in ordinary people and optimizes the function of the attention control area (DLPFC) [15]. Tai Chi promotes the functional connection of the motor area (MC), the prefrontal lobe (PFC), and the occipital lobe (OL) in ordinary people. Compared with the control group, people who received Tai Chi training had greater brain activity, including in the MC, PFC, and OL, during the rest and exercise states, enhancing the synergy of neural activities in bilateral brain regions [15, 16]. These findings prove that Tai Chi exercise activates the frontal-parietal lobe region [17]. Researchers have also found that Tai Chi promotes activation of upper limb muscles and improves proprioception. That is, it promotes downward motor output and upward sensory input. Tai Chi can activate upper limb muscles, such as the anterior deltoid and triceps brachii. Older adult Tai Chi practitioners show greater activation of the anterior and middle deltoids in reaching objects and faster reaction and exercise times [18]. Tai Chi improves the proprioception of stroke patients, indicating that the sensorimotor circuit is regulated by Tai Chi [19]. Tai Chi improves the movement pattern, increases shoulder movement, and improves the ability of movement control [20]. In addition, relevant studies have reported on how Tai Chi improves upper limb control in older adults. The results have shown that compared with control individuals, Tai Chi practitioners have a better response and exercise time, hand-eye coordination, and upper limb movement control in more complex tasks [21]. In addition, rehabilitation training promotes the coupling of the sensorimotor frontal-parietal cortex and upper limb muscle patterns to promote regular muscle coordination and reduce compensatory movement [22–28].

Based on the available data, Tai Chi improves upper limb function of stroke patients. In stroke patients, the abnormal two-way coupling of the sensorimotor frontal-parietal network and upper limb muscle activation pattern leads to abnormal upper limb muscle coordination and movement. Tai Chi can activate brain areas related to sensorimotor function and the frontal-parietal lobe, improve the activation of upper limb muscles in stroke patients, and promote sensory input. In this study, stroke patients with upper limb dysfunction will be treated with Tai Chi, and the changes in their kinematic characteristics after Tai Chi training will be evaluated based on a markless sensing technique to distinguish between recovery and compensation. This study will use multimodal Kinect, functional near-infrared spectroscopy (fNIRS), electroencephalography (EEG) and surface electromyography (EMG). Closed-loop structure and graph theory will be used to analyse the results and to clarify the cortical-muscular network functional reorganization mechanism of Tai Chi on upper limb function.

At present, domestic and foreign studies have explored the impact of different rehabilitation interventions on the brain-muscle coupling of upper limb function in post-stroke patients, such as botulinum toxin combined with rehabilitation [29], virtual reality muscle computer interface [22], transcranial direct current stimulation [30] and transcranial magnetic stimulation [31]. However, no literature reports the bidirectional interaction and reorganization mechanism of post-stroke patients’ cortical and upper limb muscle networks after Tai Chi training. Previous studies on the effects of Tai Chi after stroke have mainly focused on the overall efficacy of upper limb function and neural excitability. The results of this trial will help provide a theoretical basis for the clinical optimization of Tai Chi to improve upper limb motor function in post-stroke patients.

## Methods/design

### Study design

**Fig. 1:**
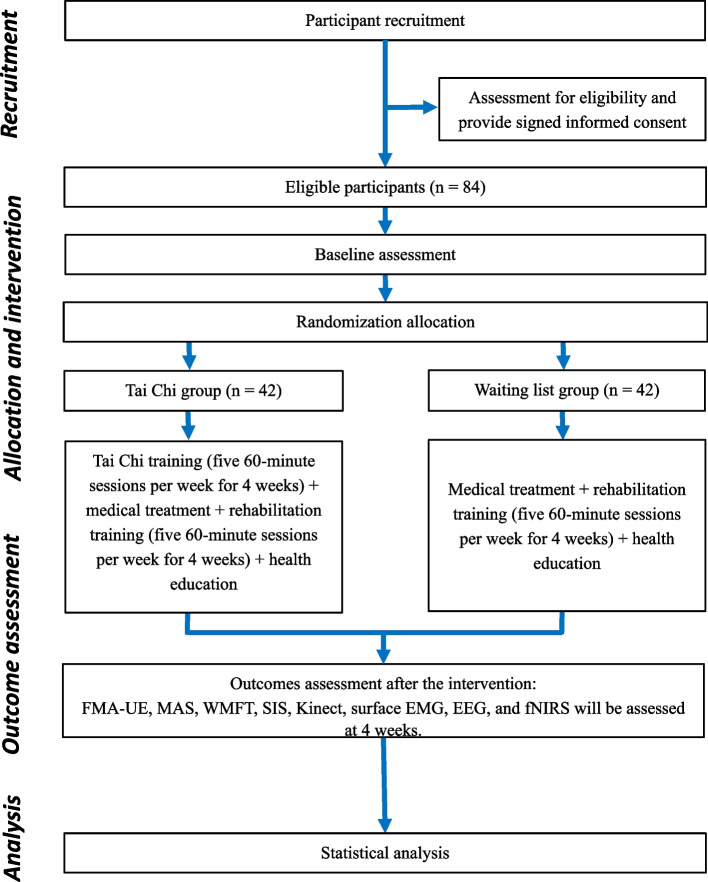
Flow diagram of the participants. Abbreviations: EEG, electroencephalography; EMG, electromyography; fNIRS, functional near-infrared spectroscopy; FMA-UE, Fugl-Meyer Upper Extremity; Kinect, Azure Kinect kinematic analysis; MAS, Modified Ashworth Scale; SIS, Stroke Impact Scale; WMFT, Wolf Motor Function Test

**Fig. 2:**
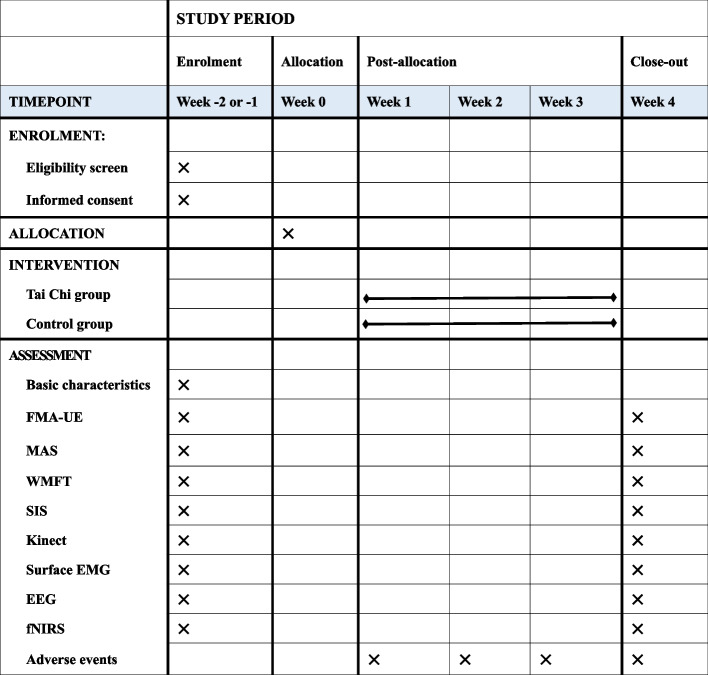
SPIRIT figure showing the enrolment, intervention, and assessment schedule. Abbreviations: EEG, electroencephalography; EMG, electromyography; fNIRS, functional near-infrared spectroscopy; FMA-UE, Fugl-Meyer Upper Extremity; Kinect, Azure Kinect kinematic analysis; MAS, Modified Ashworth Scale; SIS, Stroke Impact Scale; WMFT, Wolf Motor Function Test

A randomized, single-blind, parallel-controlled trial will be conducted to evaluate the effectiveness and neural mechanism of traditional Tai Chi training in stroke patients with upper limb dysfunction. The framework of this trial is a superiority trial design. The evaluators and data analysts will be blinded regarding the treatment. A total of 84 eligible participants will be randomly assigned to the Tai Chi group or the control group in a 1:1 ratio. Members of the Tai Chi group will receive 4 weeks of Tai Chi training five times a week, with each session lasting for 60 min (a total of 20 sessions). Participants in the control group will not receive Tai Chi training. Both groups will receive medical treatment and routine rehabilitation training. The primary and secondary outcomes will be measured at baseline and 4 weeks (the end of intervention). This study will use the SPIRIT reporting guidelines [32] (see Additional file 3 for more details). The flow chart of the research design is shown in Fig. 1, and the enrolment, intervention and assessment schedule is shown in Fig. 2. Additional file 4 shows additional details.

### Sample size estimation

This trial will observe the effects of Tai Chi on upper limb function of post-stroke patients. When calculating the required sample size using the Gpower V.3.1.9.2 software, the Fugl-Meyer Assessment Upper Extremity (FMA-UE) scale served as the primary outcome measure. Based on a published study [33], the FMA-UE scores of stroke patients in the Tai Chi and control groups after intervention were 60.50±2.19 and 59.14±1.77 points, respectively. According to these results, a sample size of 64 participants would be sufficient to detect the target effect size (0.6830377) with a type 1 error of 5% (*α* = 0.05) and 80% power (*β* = 0.20). Assuming a dropout rate of 20%, a total of 84 participants are necessary, with 42 participants in each group.

### Participants and recruitment

Eighty-four participants will be recruited from the rehabilitation hospital affiliated with the Fujian University of Traditional Chinese Medicine in Fuzhou, China. Participants will be recruited by distributing leaflets, posters and recruitment information on WeChat or other online platforms. Interested volunteers can contact the research assistant, who will screen applicants according to the inclusion and exclusion criteria. The research assistant will provide the consent form to each participant (see Additional file 5). This form provides fundamental information about the research, including the goals, the inclusion and exclusion, and potential adverse events. At the same time, the research assistant will explain that each participant will be randomly assigned to the control or Tai Chi group. Each participant must agree that if they are randomly assigned to the control group, he/she will not participate in Tai Chi. Eligible volunteers will be invited to join the study, sign the informed consent form, and then arrange the baseline assessment.

#### Inclusion criteria

To be eligible to participate in the study, each participant must meet the following criteria:

1. Meet the diagnostic criteria of ‘stroke’ in the diagnostic essentials of various cerebrovascular diseases adopted at the Fourth National Cerebrovascular Disease Academic Conference in 1995 and confirmed by head computed tomography or magnetic resonance imaging;
2. The first stroke occurred between 2 weeks and 6 months prior to commencing the study;
3. Unilateral cortical lesion (left or right hemisphere) or subcortical lesion involving the motor pathway;
4. The muscle strength of the upper limb of the affected side is ≥ 3 and muscle tone is ≤ 2 based on the Modified Ashworth Scale (MAS);
5. Right-handedness before stroke;
6. Age between 50 and 70 years;
7. Blood pressure stable and below 160/100 mmHg;
8. A Mini-Mental State Examination (MMSE) score of > 17 points for illiterate participants, > 20 points for participants with a primary school education, and > 24 points for participants with a middle school and above education;
9. Brunnstrom stage ≥ IV in the affected upper limb;
10. Standing balance ≥ level 2, the ability to stand independently for more than 5 min, and the ability to walk independently for more than 6 m;
11. Willing to sign the informed consent form and understand, accept, and implement the rehabilitation guidance.

#### Exclusion criteria

The exclusion criteria for participants were:

1. Upper limb motor dysfunction caused by other diseases such as brain tumours, brain injury, or parasitic brain disease;
2. Diseases affecting the ability to participate in Tai Chi training: serious lower limb joint diseases, arthritis, joint injury, cervical spondylotic myelopathy, lumbosacral spinal canal stenosis, and lower limb neuropathy;
3. Severe complications of stroke, such as severe pulmonary infection, shoulder hand syndrome, or venous embolism of lower limbs;
4. Severe heart disease; heart, liver, and kidney failure; malignant tumours; and gastrointestinal bleeding;
5. MMSE score ≤ 17 points for illiterate participants, ≤ 20 points for participants with a primary school education, and ≤ 24 points for participants with a middle school and above education;
6. Severe visual impairment and cannot complete the training;
7. Sensory aphasia (unable to understand the instructions);
8. Participation in Tai Chi training within the past 6 months;
9. Contraindications for near-infrared functional imaging and EEG, such as skin infection, scalp wound, dermatitis, and metal implants under electrodes;
10. Skin or muscle lesions of extremities that affect surface EMG;
11. Unable to cooperate or not suitable to participate in the examination, evaluation, and treatment of this study for other reasons, such as intolerable pain, abnormal mental state, or limited ability to move;
12. Participation in other clinical studies.

#### Dropout criteria

Participants will be withdrawn from the trial for the following reasons:

1. Subjects who experience severe adverse events and are not suitable for the trial;
2. Subjects who suffer another stroke;
3. Subjects who voluntarily withdraw from the trial;
4. Subjects with deterioration or severe complications and who need emergency intervention measures.

### Randomization and allocation concealment

After baseline assessment, eligible participants will be randomly assigned to the Tai Chi group or the control group in a 1:1 ratio. Random assignment sequences will be generated by independent statisticians using the SAS 9.1 statistical software. Opaque, sealed envelopes will hide the randomization sequence, and participants will be managed by an independent research assistant who will know nothing about recruitment, evaluation, and intervention. The independent research assistant will inform the eligible participants of their assignment results by telephone.

### Blinding

In this study, it will be impossible to blind the Tai Chi coaches and participants regarding the treatment. However, all statisticians and evaluators will be blind regarding the group assignments. This study will use a two-level blinding method: the first level uses the letters ‘A’ and ‘B’ to represent the group to which the participant is assigned, and the second level uses the letters ‘A’ and ‘B’ to represent the intervention measures, namely Tai Chi and control. At the end of the study, the data will be entered into the database according to the participant’s codes. After data entry, the database will closed and primary unblinding will be performed to determine which group of participants are assigned to ‘A’ and ‘B’. After completing all data analysis, secondary unblinding will be performed to determine whether the subjects are assigned to the Tai Chi or the control group.

### Intervention

#### Control group

The control group will receive routine medical treatment, health education, and rehabilitation training. According to the 2014 guidelines for the secondary prevention of ischaemic stroke and transient ischaemic attack in China, formulated by the cerebrovascular disease group of the neurology branch of the Chinese Medical Association in 2015, the subjects will receive routine medical care, including aetiological treatment, blood pressure management, antiplatelet therapy, anticoagulation therapy, essential treatment of heart disease, hyperglycaemia treatment, and blood glucose and lipid management. According to published guidelines [34], the following health education will be given to subjects: (1) emphasize the severe harm of stroke so that the patients can consciously take active preventive measures; (2) the principal risk factors and inducing factors of stroke and how to carry out secondary prevention; and (3) coping with stroke and rehabilitation. The therapist will conduct rehabilitation training for the subjects according to the guidelines [35]. The participants will receive rehabilitation treatment by trained occupational therapists for the affected upper limb; it will consist of five 60-min sessions per week, for a total of 20 total sessions.

During the entire 4-week intervention, all subjects will record the duration of activities each day (classified as static work, low-intensity activity, medium-intensity activity, and high-intensity activity), their diet, and the drugs they take.

#### Tai Chi group

This group will receive Tai Chi training, routine medical care, health education, and rehabilitation training. The routine care, education, and rehabilitation training will be the same as described for the control group. The Tai Chi training will be led by Tai Chi coaches with more than 5 years of qualification. Tai Chi training follows a gradual process from part to whole and from easy to complex. The teacher will choose the appropriate form of Tai Chi according to each participant’s ability. When adjusting Tai Chi training for each participant, two fundamental principles are used: (1) the participant should be able to practice Tai Chi while relaxing and (2) there should be as much overall coordination as possible. The participant relaxes his/her muscles and joints as much as possible and focuses on movements that help him/her relax, completing these movements with minimal effort. The movement is slow, repetitive, and, if necessary, carried out in sections. Eight Tai Chi poses will be adopted in this study: Starting Posture, Parting the Wild Horse’s Mane on both sides, Brush Knee and Twist Step on both sides, Forearm Rollings on both sides, Grasp the Bird's Tail – Left Side, Grasp the Bird's Tail – Right Side, Cloud Hands, and Closing Form. These poses have been selected based on the literature [4, 36]. Please refer to 24 simplified standard movements of Tai Chi promoted by the General Administration of Sport (http://www.sport.gov.cn/). In the actual training process, the accuracy and consistency of the posture routine will not be emphasized. At the same time, according to the actual movement ability of the patient, the difficulty of reaching the posture standard will be appropriately reduced. The participants will receive five 60-min sessions per week, for a total of 20 sessions. Each session will include 5 min of warm-up, 50 min of Tai Chi training, and 5 min of cooldown. The training will be conducted at Fujian provincial rehabilitation hospital. The researchers involved in this trial will not carry out relevant concomitant care and interventions that are permitted or prohibited during the trial.

### Outcome measurements

The variables of this trial include basic characteristics, primary and secondary outcomes, and exploratory results. The basic characteristics will be determined from a questionnaire administered at baseline (1–2 weeks before randomization). The primary and secondary outcomes will be measured at baseline and at the end of the intervention (4 weeks after randomization). The exploratory results will be measured at baseline and at the end of the intervention (4 weeks after randomization). All results will be assessed by experienced assessors who do not know the group assignments of the participants.

#### Primary outcome measures

The primary outcome measure is the change in the FMA-UE scale score from baseline to the end of the 4-week intervention. The FMA-UE scale includes 33 items divided into four subscales (arm, wrist, hand, and coordination), each scored on a 3-point sequential scale [37]. Scoring is based on performing independent actions inside and outside the synergy pattern. The maximum score of 66 corresponds to undamaged motor function. The FMA-UE scale is often used to classify the severity of stroke injury, and the scale shows good reliability and validity. A FMA-UE score between 32 and 57 indicates moderate upper limb injury and an FMA-UE score between 58 and 65 indicates mild injury of the contralateral arm. FMA-UE sensation (0–12), passive range of motion, and pain (0–24) will also be assessed.

#### Secondary outcome measures

Kinematic analysis includes endpoint performance parameters, joint angle, shoulder/elbow coordination index, and trunk compensatory motion. Two Kinect motion capture systems will be used for a kinematic evaluation to analyse the three-dimensional motion of the trunk and upper limbs while reach-to-grasp-pen and Tai Chi Cloud Hand. During the measurement process, the camera unit with high spatial resolution immediately calculates the three-dimensional (3D) coordinates of the mark. The Kinect SDK automatically collects data. The captured data are transferred to MATLAB software for customized analysis. Azure Kinect records XYZ coordinate data of 25 anatomical landmarks at a sampling frequency of 30 Hz. These landmarks, which do not require markers, include the head, neck, spine, left and right shoulders, left and right elbows, left and right wrists, left and right hands, left and right fingertips, left and right thumbs, middle ridges, left and right hips, left and right knees, left and right ankles, and left and right feet. To ensure stable mark recording, Azure Kinect is placed on a tripod with a height of 114 cm, an inclination angle of 20°, and a distance of 140 cm from the far end of the table. The table and chair are 75 cm and 46 cm high, respectively. The participant sits comfortably on the chair in front of Kinect and naturally completes the tasks of reach-to-grasp-pen with their upper limbs and Tai Chi Cloud Hand at their own pace. The participant is encouraged to follow the guidance video and repeat each task five times. No additional assistance is provided to the subjects unless there are safety issues.

The Modified Ashworth Scale (MAS) will be used to evaluate the change in passive motor resistance and spasm [38]. This 6-point sequential scale will be used to measure tone in the scapular adductor, elbow, wrist, and finger flexors at rest; the score ranges from 0 (muscle tension does not increase) to 4 (the affected part is stiff during flexion or extension). A MAS score of ≥ 1 in one or more muscles indicates a spasm.

The Wolf Motor Function Test (WMFT) will be used to evaluate the functional activity of the upper limbs [39]. The WMFT consists of 15 items. Items 1–6 are simple joint movements while items 7–15 are compound functional movements. The WMFT score is determined based on the quality of all actions (0–5 points, six grades).

The Stroke Impact Scale (SIS) is used to assess the overall quality of life of post-stroke patients [40]. The assessment includes a series of self-reported questions that assess the quality of life related to strength, hand function, mobility, and activities of daily living. The instrument provides a total score from 16 points (maximum injury) to 80 points (minimum injury).

#### Exploratory outcomes measures

The functional connection of the corticomuscular closed-loop network (CLN) in the resting and task states will be assessed. This mainly includes the extended partial directed coherence (FCePDC) value between the regions of interest (ROIs) and the small world measurement of the network, such as weighted clustering coefficient, weighted shortest path, and betweenness centrality.

EEG will be acquired according to the international 10–20 system. The Neuroscan system will be used to record the left and right sensorimotor and frontal-parietal cortex EEG. EEG is recorded from 32 Ag/AgCl scalp electrodes (sampling rate of 1000 Hz) using a BrainAmp DC amplifier and an anti-aliasing filter. The electrodes are designated Fz, F4, FC5, FC3, FC1, FCz, FC2, FC4, FC6, C5, C3, C1, Cz, C2, C4, C6, CP5, CP3, CP1, CPz, CP2, CP4, CP6, P3, POz, P4, POz, O1, O2, FCZ (reference), and AFZ (ground). The electrode impedance is kept below 10 kΩ. Some of the electrodes are located on the scalp in the central frontal lobe (FC3, FC4), centre (C3, C4, C5, C6), and the central parietal lobe (CP3, CP4).

EMG will be recorded from signals from the following muscles: the bilateral superior trapezius, anterior deltoid, posterior deltoid, middle deltoid, biceps brachii, triceps brachii, extensor carpi radialis, and flexor carpi radialis. The surface EMG signal will be recorded at 1500Hz through the Noraxon system using disposable Ag-AgCl electrodes with bipolar configuration, with a 2-cm distance between electrodes. After the standard skin preparation procedure, electrode pairs will be placed on each muscle’s abdomen, and the muscle’s surface EMG will be identified by palpation and a few blank tests of elbow flexion and extension. In addition, EMG signals will be monitored online before the experiment to evaluate the positioning and recording quality of the electrodes.

fNIRS uses a continuous wave optical system to measure oxygenated haemoglobin (HbO) in the cerebral cortex. The system’s light source generates near-infrared light (690 nm and 830 nm) at a fixed sampling rate of 10 Hz and a total of 95 channels. A 3D digitizer will be used to obtain the coordinates of the channel. These coordinates will be converted into the MNI coordinates, and then the NIRS_SPM toolbox will be used to project it onto the standard MNI brain model. With the above configuration, the channel can cover the SMA, the PMC, M1, the primary sensory cortex (S1), and the DLPFC.

### fNIRS-EEG-EMG experimental paradigm

There will be three tasks: upper limb forward reach, elbow flexion and extension, and Tai Chi Yunshou. After a 3-min rest period (baseline), the motor paradigm will use a three block design:

1. Upper limb forward reach with three cycles of preparation [3 s] – reach forward [3 s] – rest [3 s];
2. Elbow flexion and extension with three cycles of preparation [3 s] – elbow flexion [3 s] – rest [3 s] – elbow extension [3 s] – rest [3 s];
3. Tai Chi Yunshou with three cycles of preparation [3 s] – Tai Chi Yunshou [3 s] – rest [3 s]

For each motor task, there will be 3 s of preparation. The subjects will be asked to perform specific motor tasks for 3 s according to the instructions and then relax for 3 s. Each motor task will consist of 20 repetitions. The tests will be carried out on both sides of the arm of post-stroke patients. Each subject will use his/her left and right hands to perform each motor task. He/she will take a 5-min break between each task to prevent muscle fatigue. The experiment will last for 30 min for each subject (2 hands × 3 tasks × 20 repetitions). Finally, the static state will be assessed for 3 min. Data will be collected at baseline and after the 4-week intervention.

### Safety evaluation

During the study intervention, any adverse events that occur the subjects will be monitored, recorded on the adverse event case report form (CRF), and then reported to the research assistant. The causal relationship between Tai Chi training and the severity of adverse events will be analysed. Adverse events are defined as any dysfunction caused by the intervention, such as knee or ankle sprain, knee pain, or hypoglycaemia. In the case of any adverse event, the coach or project manager will provide complementary treatment for the participants. Serious adverse events will be reported to the rehabilitation hospital’s ethics committee affiliated with the Fujian University of Traditional Chinese Medicine. Additional file 5 provides additional details.

The safety of subjects will be evaluated based on several factors. The evaluation results are divided into level 1 (safe, no adverse reactions); level 2 (safe but with mild adverse reactions that do not require treatment; subjects can continue to participate in training); level 3 (safety problems and moderate adverse reactions, but the subjects can continue training after receiving treatment); and level 4 (study terminated due to severe adverse reactions) (Common Terminology Criteria for Adverse Events [CTCAE] version 5.0, US Department of Health and Human Services, 2017).

### Data collection and management

The assessors will collect data using a paper CRF. The research assistant will control the quality of data collection and input these data into Microsoft Excel. Double data entry will be employed to avoid data entry errors. The entered data will be imported into the data management platform FJTCM Yun, available at http://10.252.47.2, and managed by an independent agency. All data will be treated with the highest confidentiality. If a participant withdraws from the trial, the researchers will ask the participant if they would be willing to participate in the follow-up data collection. In that case, the researchers will make every effort to obtain follow-up data for the participant.

### Statistical analyses

#### Behavioural indicators of upper limb function level

Independent statisticians who will not participate in evaluations will analyse the data using SPSS Statistics for Windows Version 24.0, with *P* < 0.05 considered statistically significant. Continuous variables with a normal distribution will be presented as the mean ± standard deviation (SD), and continuous variables with a non-normal distribution will be presented as the median and interquartile range (IQR). Categorical variables will be reported as frequency or percentage. Basic data such as age, gender, and body mass index (BMI) will be considered covariates. The primary and secondary outcomes will be analysed according to the intention-to-treat (ITT) and protocol-compliant (PP) populations. The results of the ITT analysis will be compared with the results of the PP analysis to determine whether they are consistent. Multiple imputation will be used for missing data. ITT analysis will be performed on all Tai Chi and control group subjects. After the subjects are randomly divided into groups, they will be included in the assigned group for statistical analysis, even if they withdraw from the trial due to adverse reactions. Student’s *t* test or the Mann-Whitney *U* test will be used to analyse the differences between the Tai Chi and the control groups at each time point (including at the end of the 4-week intervention). A linear mixed model with limited maximum likelihood and group × time interaction analyses will be performed. Pearson or Spearman correlation analysis will be used for normally and non-normally distributed data, respectively.

#### Kinematic data analysis

The upper limb model of the University of Western Australia (UWA) will be applied to the 3D motion analysis of the landmark motion analysis system. The model includes 18 markers; the trunk, upper arm, forearm, and hand segments are defined according to the location of anatomical markers. The calibration anatomical system technology is used to establish the markers related to the coordinate system of the upper arm group or the forearm group. The movement of the upper limb landmarks can be reconstructed from their position relative to the upper limb technical coordinate system. The 3D coordinates of the anatomical markers recognized from the Kinect system bone model are recorded during the task. Local segment coordinates are established, including the torso and upper arm; each segment coordinate is based on global coordinates.

Azure Kinect SDK2.0 and Microsoft Visual Studio 2016 will be used to save the kinematic data and time range. The 3D position data collected by the Kinect sensor will be imported into MATLAB 2018a and then processed using a method previously developed by the research team to establish the kinematics model of Azure Kinect. Azure Kinect can measure XYZ coordinate data of 25 joints of the body. Kinematic data of 10 joints, including the spine, left and right shoulder, left and right elbow, left and right wrist, and left and right hand, will be used. Each repetition segments the original data and is then filtered by singular spectrum analysis (SSA) to reduce the impact of noise. The analysis calculates the kinematic index according to the filtered data. For the Kinect upper limb evaluation system, the measured node space position is used as the input, and the developed markerless motion analysis system calculates the joint angle and space-time parameters. A Butterworth low-pass filter with a cut-off frequency of 6 Hz is used. Then, the customized upper limb kinematics calculates the three Euler angles of shoulder rotation for the Azure Kinect system, which follow the sequence of flexion (+)/extension (−), adduction (+)/abduction (−), and internal rotation (+)/external rotation (−). Elbow flexion is calculated using trigonometric functions from the position data of ShoulderRight, ElbowRight, and WristRight.

#### Analysis of fNIRS, EEG, and EMG data

For data pre-processing, a notch filter first filters the original EEG signal at a frequency of 50 Hz to eliminate power line noise, followed by a fourth-order Butterworth bandpass filter (0.5–45 Hz). Independent component analysis is used to remove eye movement artifacts. The data are re-referenced by subtracting the average of all channels from each channel. Subsequently, the EEG data are subdivided into multiple tests. These tests start from 500 ms before the tasks and last to 3000 ms after the baseline, and each test is corrected. Finally, any test with artifacts is manually checked and excluded. For fNIRS signals, a fourth-order Butterworth bandpass filter (0.01–0.5 Hz) is applied to eliminate artifacts, such as cardiac interference (0.8 Hz). Then, the wavelet-based method removes the motion artifacts from the fNIRS signal. The changes in haemoglobin (HbO) and deoxygenated haemoglobin (HbR) concentrations are calculated according to the modified Beer-Lambert law. The signals obtained from each channel are checked manually, and noisy tests are excluded from further analysis. A general linear model is used to identify activation channels that are significantly induced by each hand motion. These channels are used as a spatial prior for EEG source imaging.

Functional connectivity analysis is based on graph theory. Theoretically, the network can be represented by a graph composed of vertices and corresponding edges. From the perspective of the CLN framework, through the network method based on weighted graph theory and considering the edge strength of the FC CLN, the dynamic changes in cortical-muscular-cortical coordinated working mode before and after Tai Chi are characterized. Specifically, the correlation matrix between different electrode signals is established to define the corresponding weights. The matrix coefficients are taken as Wxy and link the X and Y points. FcePDC is used as the value. The FcePDC value of the matrix in the EEG-EEG, EEG-EMG, and EMG-EMG channel pairings of 40 vertex networks (40 EEG and 8 EMG) is used as the edge weight. Based on weighted graph theory, three global matrices are used to measure the network characteristics. The small world measurement of the network—that is, the weighted clustering coefficient and the weighted shortest path—is used to quantitatively evaluate the local efficiency of information transmission and the ability of parallel information transmission in the network. In addition, the betweenness centrality is used to analyse the regional characteristics in CLN.

Effective connectivity (EC) analysis of the CLN is based on defining brain and muscle regions and ROIs. The basis for extracting ROIs from brain regions is that the brain map guides ROIs, and there are no overlapping regions to ensure the selection of functionally meaningful connected regions. The cortical reconstruction of the head model is used as the accurate geometric head model of the subjects. The EEG signals collected from the standard electrode leads of the international 10–20 system are represented on an accurate geometric model. First, the motor areas of the unaffected and affected sides are selected as ROIs, defined as Mu and Ma, respectively. The unaffected and affected somatosensory cortices are also considered to be ROIs, corresponding to Su and Sa, respectively. Finally, ROIs from unaffected and affected frontal and parietal regions are defined as Fu, FA, PAu, and PAa, respectively. For the muscle regions related to the two anatomical parts of the upper limb, according to the signals collected from the myoelectric electrodes on the affected side, the proximal (including the superior trapezius, the anterior deltoid, the posterior deltoid, the middle deltoid, the biceps brachii and the triceps brachii) and distal muscles (including the flexor carpi radialis and the extensor carpi radialis longus) of the upper limb are divided into two regions, which are defined as PM and DM. Based on the above 14 ROIs, the inflow index (IFI) and the outflow index (OFI) are used to determine the causal relationship between ROIs before and after training. To determine the difference in the average FcePDC value between the two groups in each frequency band before and after training, the FcePDC value calculated before training is defined as the baseline. The baseline changes are calculated by subtracting the baseline before training from the post-training values, and an independent-sample two-tailed *t*-test is performed to compare the changes between the two groups. In addition, repeated measures analysis of variance is used to test the statistical difference in the average FcePDC values in different information flow directions before and after training. Bonferroni correction is used to correct for multiple comparisons. The inter-group differences in network parameters (weighted clustering coefficient [C_W_] and weighted shortest path length [L_W_]) are tested with the Mann-Whitney *U* test. For EC analysis, the IFI and OFI values of ROI changes before and after training in the two groups are tested by paired two-tailed *t*-tests. The consistently paired *t*-test is used for the betweenness centrality parameter (BCX). The confidence level *α* is defined as 0.05.

Correlation analysis will also be employed. For the above brain functional features (based on brain imaging and graph theory analysis), brain regions with significant differences between groups are selected and relevant eigenvalues are extracted. The eigenvalues of functional brain connections of brain regions with significant differences or the partial and factor correlations between the eigenvalues of functional brain connections and upper limb scores are determined. Moreover, the factors that have the most significant impact on the cortex are analysed. The results are controlled for the influence of age, gender, education level, and other factors. Because the trial uses a single-blind design, the statisticians will conduct an interim analysis according to the label but without unblinding to guide the follow-up study.

### Quality control

Before the implementation of the project, a standard research manual and CRF will be developed, and neuropsychological experts will be hired to carry out standardized training of the research evaluators so that they understand each step of the project implementation in detail. This endeavour will ensure the quality of the project. The clinical trial quality control committee will comprise one professor, two associate professors, and one doctor of the project team. The researchers will report the progress of the clinical trial to the clinical trial quality control committee every month for evaluation.

### Dissemination policy

The results will be published in peer-reviewed journals and communicated to the participants, health care professionals, and other relevant groups. All researchers and other colleagues who will participate in the future will become co-authors of this study based on their contributions.

## Discussion

The impairment of activities of daily living caused by upper limb dysfunction seriously affects the ability of post-stroke patients to return to their families, communities, and workplaces. However, there is no specific drug to treat upper limb dysfunction in clinical practice, and the reported rehabilitation training for upper limb dysfunction lacks high-quality evidence. A systematic review suggests that post-stroke patients can improve upper limb function through long-term upper limb rehabilitation training, but the medical cost of long-term upper limb rehabilitation training is high [41, 42]. Therefore, finding a safe, effective, well-defined, and low-cost rehabilitation treatment would substantially help patients return to society and reduce medical costs.

The recovery process of upper limb function is considered to require recovery of coupling the sensorimotor frontoparietal network and the upper limb muscle activation mode. Tai Chi represents an effective training method that can improve upper limb function of post-stroke patients. We propose that Tai Chi promotes the two-way sensorimotor control circuit of the cortex and muscle to promote regular muscle coordination and movement patterns to recover upper limb function. Can Tai Chi reduce the compensatory pattern to improve upper limb function after a stroke? Does Tai Chi promote the interaction between the sensorimotor frontal-parietal network and muscle activation patterns, thus promoting regular muscle coordination and movement patterns and reducing compensatory activities?

We will use several methods to improve the quality of this trial. Senior physical education teachers will act as Tai Chi coaches to ensure the quality of the Tai Chi training. The participants in the Tai Chi group will gather at a fixed time and place to practice. To control deviation caused by the amount of exercise, all participants will be required to keep an activity log. In addition, Tai Chi training will be supervised by two research assistants to ensure that both coaches and participants practice carefully. However, this trial may have some potential limitations. First, it is difficult for people involved in non-drug randomized controlled trials to be blind to the treatment. Indeed, in this study neither the coaches nor the participants can be blind to the treatment. Tai Chi coaches cannot participate in this study’s recruitment, evaluation results, or data analysis, and performance differences may be inevitable. Second, there is no follow-up. Finally, all participants will come from the same city, reducing the sample’s representativeness.

Tai Chi emphasizes the ‘unity of form and spirit’. When the ‘movement intention’ is generated, the corresponding brain regions are excited, and the resulting nerve impulses excite the muscle groups, thus completing the ‘voluntary movement’. This is the process of ‘the mind following the movement’ [43]. Through Tai Chi exercise, we can regulate the body, mind, and collaterals to promote recovery of upper limb function in post-stroke patients. This project will focus on the connection between the neural network (sensorimotor frontoparietal network) and the muscle network; consider the brain plasticity theory; and employ advanced research techniques such as fNIRS, EEG, and EMG to investigate the mechanism by which Tai Chi promotes functional recovery of upper limbs after stroke. This trial will advance the field, as the available research on the effect of Tai Chi on upper limb function after stroke has mainly focused on the overall effect on upper limb function and nerve excitability. These studies have not reported the mechanism for the interaction between the sensorimotor frontal-parietal cortex network and upper limb muscle activation mode of upper limb function.

A statement jointly released by the American Heart Association and the American Stroke Association predicts that by 2030, the United States’ expenditure on stroke and related disorders will increase to $240.67 billion [44]. Tai Chi has potential benefits for recovering upper limb motor function and daily living ability in post-stroke patients. At the same time, Tai Chi is a low-cost exercise, can save medical resources, reduces the economic burden on society and families, and has good economic benefits. In addition, this project provides evidence support for the rehabilitation effect of Tai Chi through high-quality randomized controlled trials and exploratory mechanisms research, which is conducive to the application and promotion of Tai Chi in hospitals, communities, and families.

In conclusion, this study is the first randomized controlled trial to systematically evaluate the effect of Tai Chi on the upper limb function of post-stroke patients from the perspective of neurophysiology. Significant results can provide rigorous evidence and a theoretical basis for applying Tai Chi training to post-stroke patients with upper limb dysfunction. The results will help understand the potential for adaptive brain reorganization and neuromuscular motor control mechanism, provide a reliable theoretical and experimental basis for the clinical application of Tai Chi, and provide a valuable reference for future research regarding how Tai Chi effectively improves upper limb function after stroke.

## Trial status

The first version of this trial was approved on March 4, 2022. The trial started on March 15, 2022. We hope to achieve our research objectives by December 2024.

## Protocol amendments

If there are any protocol amendments, the sponsor will be notified first, and then the principal investigator (PI) will notify the centre. The modified copy of the agreement will be sent to PI and added to the file on the researcher’s website. Any breach of protocol in this study will be fully recorded using the default report form, and the protocol will be updated in the clinical trial registry.

### Supplementary Information

**Additional file 1.** Ethical approval document.

**Additional file 2.** Funding documentation.

**Additional file 3.** SPIRIT checklist.

**Additional file 4.** SPIRIT Figure & Flow diagram.

**Additional file 5.** Model consent form.

## Data Availability

In order to protect the privacy of participants, the data set generated or analysed during this study will not be made public. However, it can be obtained from the corresponding author upon reasonable request.

## Abbreviations

EEG: Electroencephalography
EMG: Electromyography
FcePDC: Extended partial directed coherence
FMA-UE: Fugl-Meyer Assessment of the Upper Extremity
fNIRS: Functional near-infrared spectroscopy
IFI: Inflow index
MAS: Modified Ashworth Scale
MMSE: Mini-Mental State Examination
OFI: Outflow index
WMFT: Wolf Motor Function Test
SIS: Stroke Impact Scale
