# Supplementary material for: Neurobiomechanical mechanism of Tai Chi to improve upper limb coordination function in post-stroke patients: a study protocol for a randomized controlled trial
Source: Trials. 2023 Dec 4;24:788. doi: 10.1186/s13063-023-07743-w (PMC10696787; doi:10.1186/s13063-023-07743-w)

| **Ethical Review Opinions** | | | |
| --- | --- | --- | --- |
| Comment No | 2021KY-005-03 | | |
| Project name | Study on the neuromechanical mechanism of Taijiquan in improving upper limb motor strategies in stroke patients | | |
| Project source | The doctoral program of Fujian University of Traditional Chinese Medicine | | |
| Research unit | Fujian University of Traditional Chinese Medicine | | |
| Main researchers | XIE Qiurong | | |
| Review category | Review of amendments | Review method | Meeting review |
| Review date | March 4, 2022 | Review location | Rehabilitation hospital affiliated with Fujian University of Traditional Chinese Medicine |
| Review committee | He Jian, Wang Xin, ye Huaqiang, Xiao Jianping, Wei Songqing, Huang saie, Chen Shuijin, Yang Shangwang, Xie Liyu, Zhong Weihong | | |
| Review documents | The revised clinical research protocol | Version No.: 3.0 Version date: 2022-02-22 | |
|  | Revised informed consent | Version No.: 3.0 Version date: 2022-02-22 | |
|  | Revised recruitment materials | Version No.: 3.0 Version date: 2022-02-22 | |
| **Review comments**  According to ethical principles of The measures for ethical review of biomedical research involving human beings issued by the National Health Commission, The code for ethical review and management of clinical research of traditional Chinese Medicine issued by the State Administration of Traditional Chinese Medicine, The State Food and Drug Administration "clinical trial quality management specifications for drugs" and "clinical trial quality management specifications for medical devices," Helsinki advice of the World Medical Association, International ethical guidelines for biomedical research in human bodies, International Committee of Medical Sciences. After review by the ethics committee, the opinions are as follows:  Agree to modify "clinical research protocol, informed consent, and materials for recruiting subjects." | | | |
| Adjusted annual/periodic follow-up review frequency | Please submit the research progress report within one month on March 4, 2023 | | |
| Ethics committee | Ethics Committee of rehabilitation hospital affiliated with Fujian University of Traditional Chinese Medicine | | |
| Signature of chairman | He Jian | | |
| Date | March 4, 2022 | | |


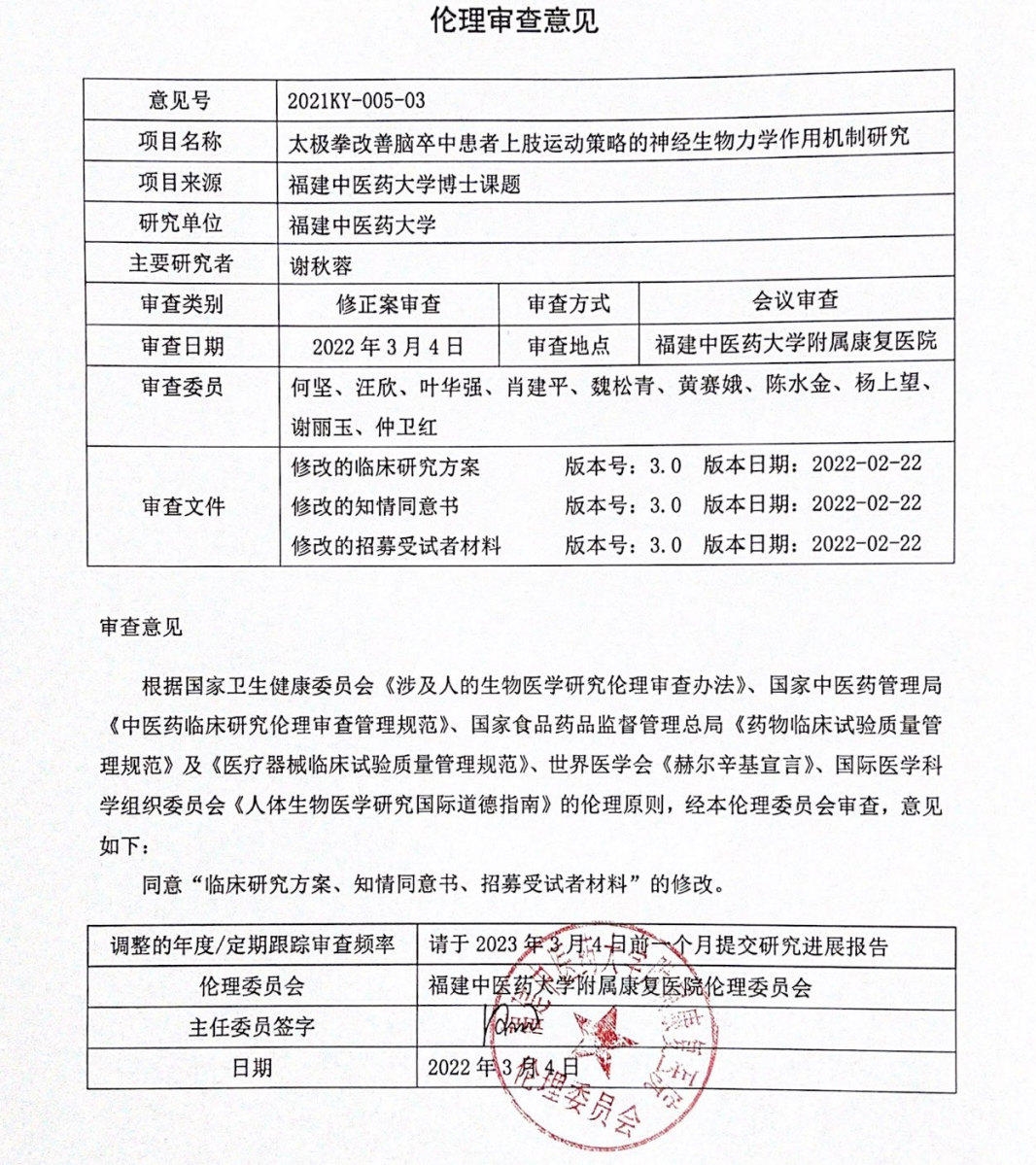

Supplement: Supplementary file 1 — Additional file 1. Ethical approval document. [file 13063_2023_7743_MOESM1_ESM.docx]
