# Supplementary material for: Neurobiomechanical mechanism of Tai Chi to improve upper limb coordination function in post-stroke patients: a study protocol for a randomized controlled trial
Source: Trials. 2023 Dec 4;24:788. doi: 10.1186/s13063-023-07743-w (PMC10696787; doi:10.1186/s13063-023-07743-w)

**Funding Documentation**

**1. the National Natural Science Foundation of China (82305357)**

| **Notice on approval of projects supported by the National Natural Science Foundation of China**  **(lump sum project)**  Mr. / Ms.:  Xie Qiurong  Following the regulations of the National Natural Science Foundation of China, relevant project management measures, and expert evaluation opinions, the National Natural Science Foundation of China (from now on referred to as NSFC) decided to fund the project you applied for. Project approval No.: 82305357, project name: Study on the mechanism of Tai Chi to improve upper limb motor function after stroke based on the coupling of sensorimotor - frontoparietal cortex and muscle activation mode, funding: 300,000 CNY, project start and end date: January 2024 to December 2026. The review comments and modification comments on the project are attached. Please log in to the NSFC network information system (https:/isisn.nsfc.gov.cn) immediately. Carefully read the instructions for filling in the plan of projects supported by the National Natural Science Foundation of China and fill in the program of projects supported by the National Natural Science Foundation of China (from now on, referred to as the plan) as required. For the items with modification opinions, please adjust the relevant contents of the project in time according to the modification opinions. If you have any objection to the modification opinions, you must submit it to the appropriate scientific department before the deadline for submission of the electronic version of the plan.  Please submit the electronic version of the plan through the NSFC network information system (https:/isisn.nsfc.gov.c) and submit it to the NSFC after being reviewed by the supporting unit. Those who fail to pass the review of the NSFC will modify the returned electronic version of the plan and then submit it. Those who pass the assessment will print the paper version of the program (in duplicate, double-sided printing) and sign in the commitment column of the project leader. The supporting unit shall affix the official seal of the supportive team to the commitment column and attach the application's paper signature and seal page to one of the plans, which shall be submitted to the project material receiving a working group of NSFC. The paper version of the plan shall be consistent with the electronic version approved. NSFC will review the application's paper signature and seal page and allow the relying unit to modify or supplement the toilet.  The deadline for submitting the electronic version of the plan to NSFC, submitting the paper version of the plan, and submitting the paper signature and seal page of the application is as follows:  1. 16:00 on September 7, 2023: the deadline for submitting the electronic version of the plan (regarded as the formal submission time of the plan);  2. 16:00 on September 14, 2023: the deadline for submitting the revised electronic version of the plan:   1. September 21, 2023: the deadline for submitting the paper version of the plan (one of which contains the paper signature and seal page of the application). 2. October 7, 2023: Deadline for submitting the paper signature and stamp page of the revised application. |
| --- |


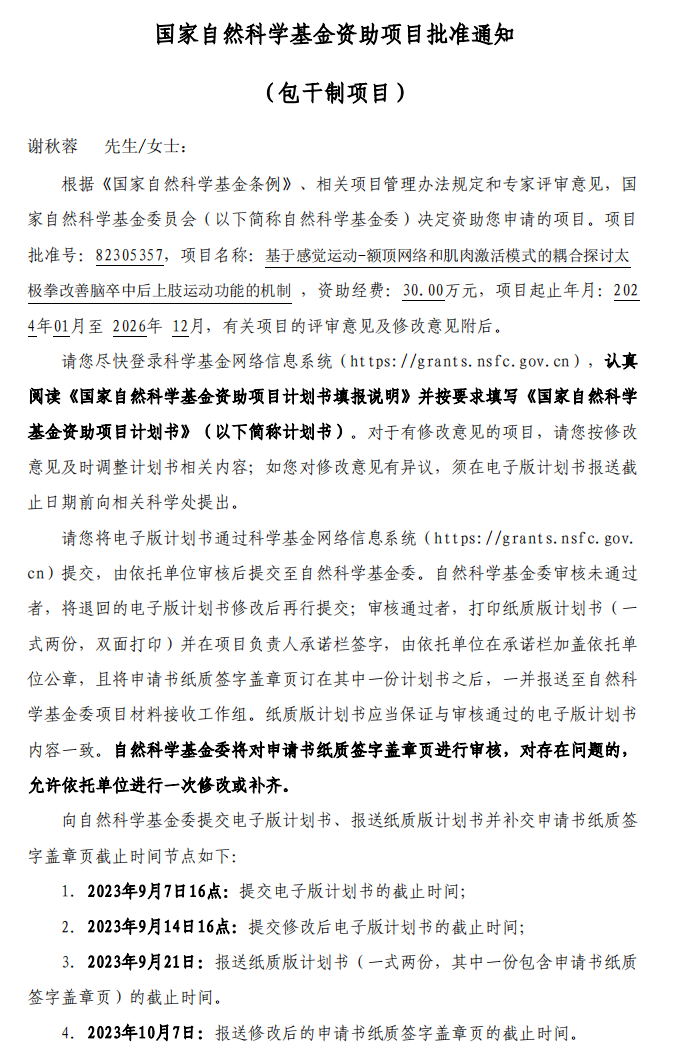


**2. Natural Science Foundation of Fujian Province (2020J01752)**

| **Fujian Science and Technology Project Management Information System**  **[(fujian.gov.cn)](http://xmgl.kjt.fujian.gov.cn/showLoginPage.do?type=fujian&loginflag=false)** | | | | | | |
| --- | --- | --- | --- | --- | --- | --- |
| **User Name：**  **XIE Qiurong** |  | **Funding year** | **Financial document No** | **Project number** | **Project name** | **Project type** |
|  | 1 | 2020 | Grant No. of Fujian Scientific Research Office（2020）29 | 2020J01752 | Artificial intelligence-based upper limb motor function evaluation and rehabilitation training system for stroke patients | Natural Science Foundation Project |


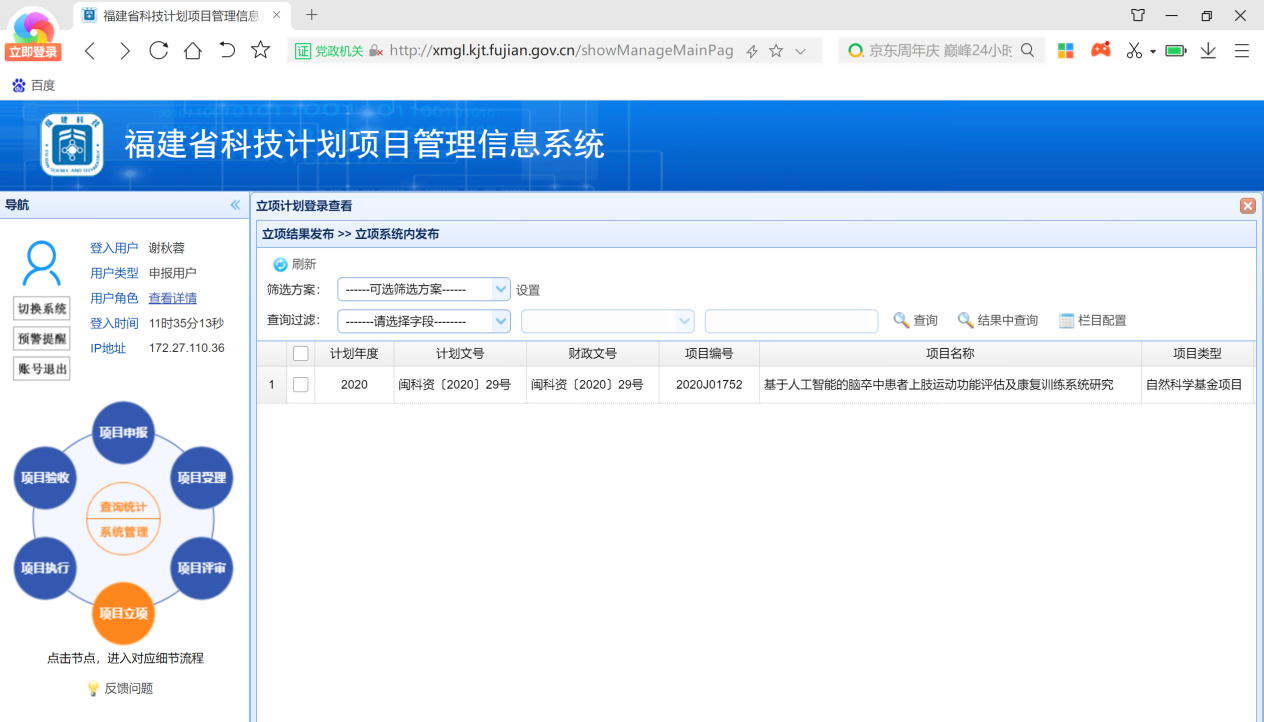


|  | **Plan document No：** Grant No. of Fujian Scientific Research Office（2020）29  **Category of planned funds：** Natural Science Foundation  **Project type：** Natural Science Foundation Project  **Project number：**2020J01752 |
| --- | --- |
| **Natural Science Foundation of Fujian Province**  **Project Duty Book**  **Project name：** Artificial intelligence-based upper limb motor function evaluation and rehabilitation training system for stroke patients  **Project undertaking unit：** Rehabilitation Medical College of Fujian University of Traditional Chinese Medicine  **Project implementation management organization：** Fujian University of Traditional Chinese Medicine  **Project leader：** XIE Qiurong  **Mobile phone number：**15005000559  **Project start and end time：**2020-11-01 to 2023-11-01  **Fujian Provincial Department of Science and Technology** | |


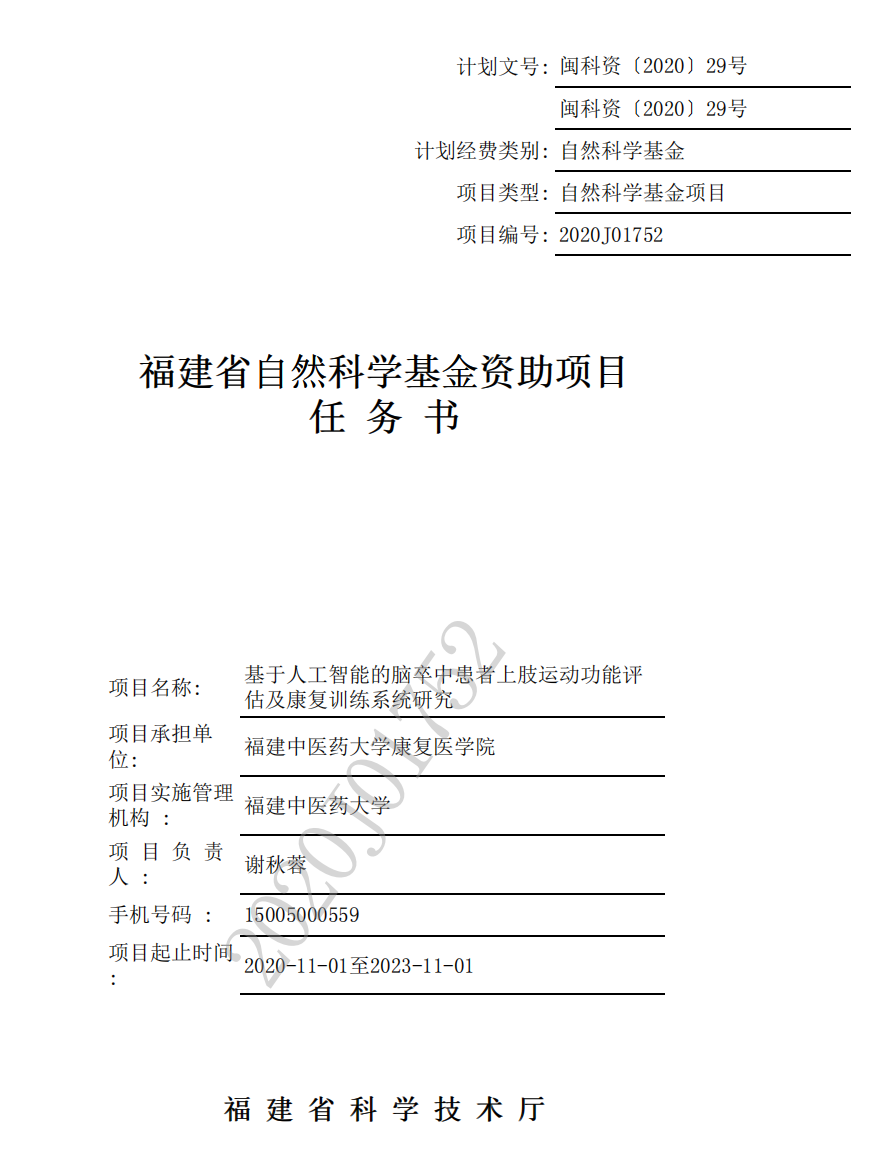


1. **the National Natural Science Foundation of China under Grants (62103252)**

| **Notice on approval of projects supported by the National Natural Science Foundation of China**  **(lump sum project)**  Mr. / Ms.:  Sheng Bo  Following the regulations of the National Natural Science Foundation of China, relevant project management measures, and expert evaluation opinions, the National Natural Science Foundation of China (from now on referred to as NSFC) decided to fund the project you applied for. Project approval No.: 62103252, project name: Research on Intelligent rehabilitation evaluation of stroke patients driven by multimodal electromechanical data, funding: 30 million yuan, project start, and end date: January 2022 to December 2024. The review comments and modification comments on the project are attached. Please log in to the NSFC network information system (https:/isisn.nsfc.gov.cn) as soon as possible. Carefully read the instructions for filling in the plan of projects supported by the National Natural Science Foundation of China and fill in the plan of projects supported by the National Natural Science Foundation of China (from now on, referred to as the plan) as required. For the items with modification opinions, please adjust the relevant contents of the plan in time according to the modification opinions: if you have any objection to the modification opinions, you must submit it to the relevant scientific department before the deadline for submission of the electronic version of the plan.  Please submit the electronic version of the plan through the NSFC network information system (https:/isisn.nsfc.gov.c) and submit it to the NSFC after being reviewed by the supporting unit. Those who fail to pass the review of the NSFC will modify the returned electronic version of the plan and then submit it. Those who pass the review will print the paper version of the plan (in duplicate, double-sided printing) and sign in the commitment column of the project leader. The supporting unit shall affix the official seal of the supporting unit to the commitment column and affix the application's paper signature and seal page to one of the plans, which shall be submitted to the project material receiving working group of NSFC. The paper version of the plan shall be consistent with the electronic version approved. NSFC will review the application's paper signature and seal page and allow the relying unit to modify or supplement the toilet.  The deadline for submitting the electronic version of the plan to NSFC, submitting the paper version of the plan, and submitting the paper signature and seal page of the application is as follows:  1. 16:00 on October 22, 2021: the deadline for submitting the electronic version of the plan (regarded as the formal submission time of the plan);  2. 16:00 on October 29, 2021: the deadline for submitting the revised electronic version of the plan:  3. 16:00 on November 5, 2021: the deadline for submitting the paper version of the plan (one of which contains the paper signature and seal page of the application) |
| --- |


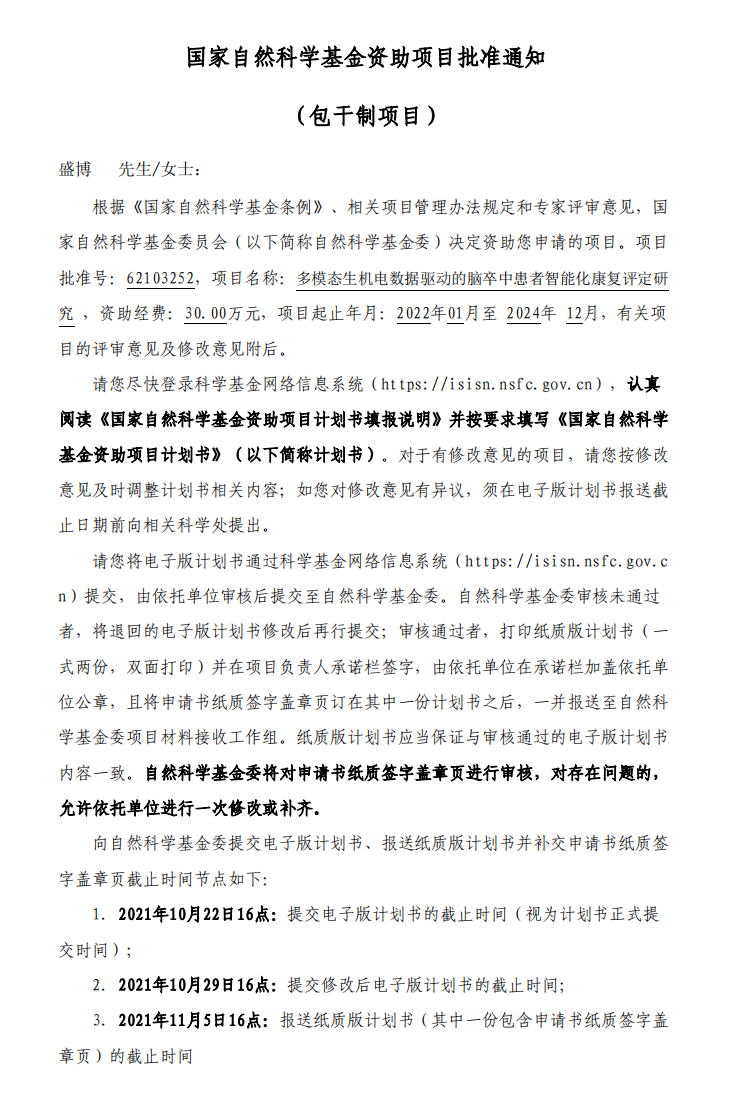


1. **the Shanghai Pujiang Program (21PJ1404000).**

| **Certificate of Honor** | |
| --- | --- |
| 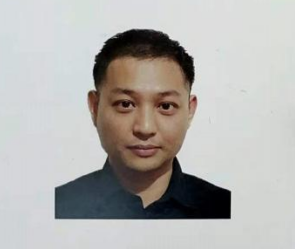  **Project Number:21PJ1404000** | **Mr. Sheng Bo:**  **He was selected as the candidate for Shanghai Pujiang (Class A) talent plan in 2021.**  **Shanghai Municipal Commission of Science and Technology**  **November 2021** |


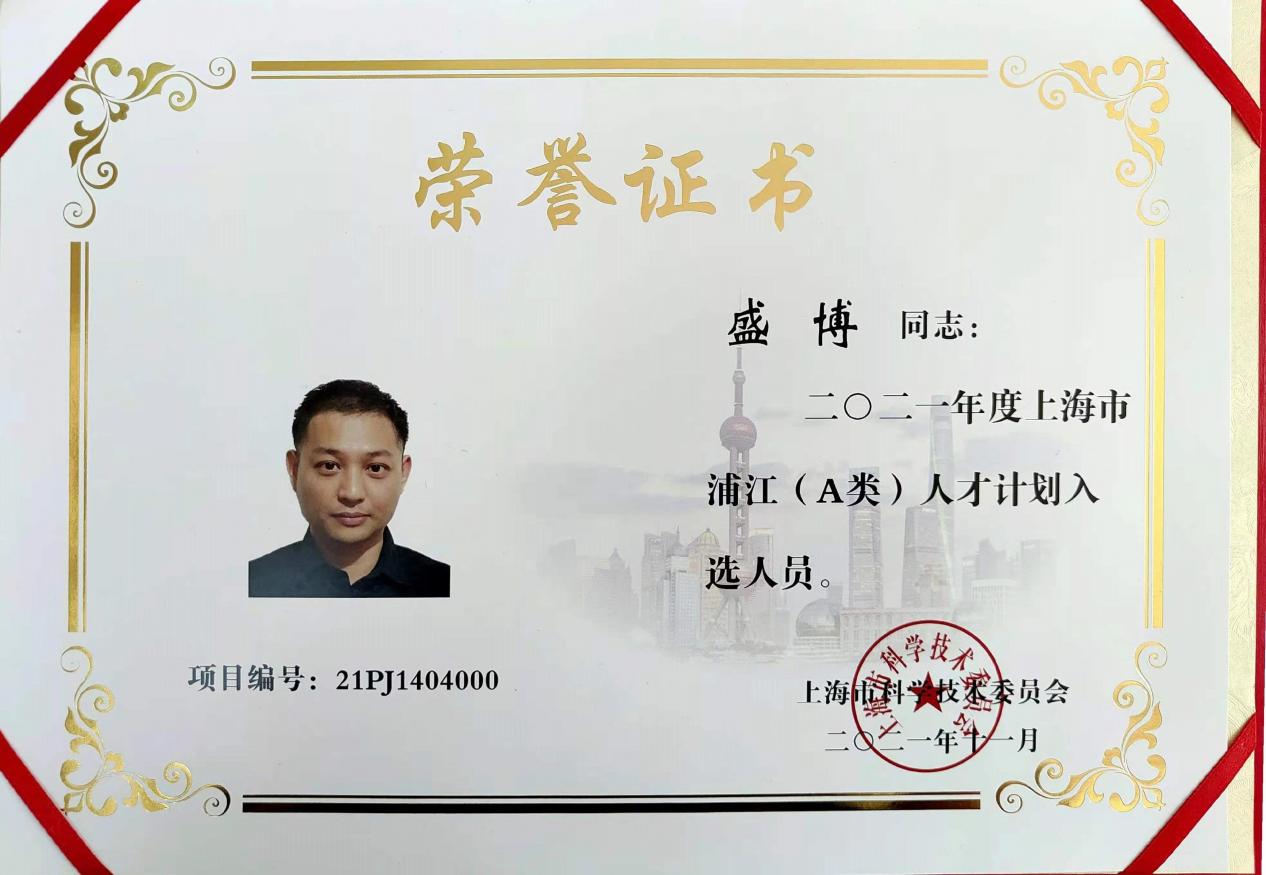

Supplement: Supplementary file 2 — Additional file 2. Funding documentation. [file 13063_2023_7743_MOESM2_ESM.docx]
