# Supplementary material for: Neurobiomechanical mechanism of Tai Chi to improve upper limb coordination function in post-stroke patients: a study protocol for a randomized controlled trial
Source: Trials. 2023 Dec 4;24:788. doi: 10.1186/s13063-023-07743-w (PMC10696787; doi:10.1186/s13063-023-07743-w)
Supplement: Supplementary file 4 — Additional file 4. SPIRIT Figure & Flow diagram. [file 13063_2023_7743_MOESM4_ESM.docx]

**Figures**

**Figure.1 Flow diagram of participants.**

***Recruitmen***

Statistic analysis

Participants recruitment

Assessment for eligibility and signed informed consent

Eligible participants (n=84)

Baseline assessment

Randomization allocation

Tai Chi group (n=42)

Waiting list group (n=42)

Tai Chi training (five 60-minute sessions per week for 4 weeks) + medical treatment + rehabilitation training (five 60-minute sessions per week for 4 weeks) + health education

Outcomes assessment after intervention:

FMA-UE, MAS, WMFT, SIS, Kinect, sEMG, EEG, fNIRS will be assessed at 4weeks.

***Allocation and intervention***

***Outcomes assessment***

***Analysis***

Medical treatment + rehabilitation training (five 60-minute sessions per week for 4 weeks) + health education

**Fig.1** Flow diagram of participants. Abbreviations: FMA-UE, The Fugl-Meyer assessment of the upper extremity; MAS, Modified Ashworth Scale; WMFT, Wolf motor function test; SIS, Stroke Impact Scale; Kinect, Azure Kinect kinematic analysis; sEMG, Surface electromyography; EEG, Electroencephalogram; fNIRS, functional near-infrared spectroscopy

**Figure. 2 SPIRIT figure. Schedule of enrolment, interventions, and assessments**

|  | **STUDY PERIOD** | | | | | |
| --- | --- | --- | --- | --- | --- | --- |
|  | **Enrolment** | **Allocation** | **Post-allocation** | | | **Close-out** |
| **TIMEPOINT** | **week-2-(-1)** | **0** | **week 1** | **week 2** | **week 3** | **week 4** |
| **ENROLMENT:** |  |  |  |  |  |  |
| **Eligibility screen** | **×** |  |  |  |  |  |
| **Informed consent** | **×** |  |  |  |  |  |
| **ALLOCATION** |  | **×** |  |  |  |  |
| **INTERVENTIONS:** |  |  |  |  |  |  |
| **TaiChi group** |  |  |  |  |  |  |
| **Control group** |  |  |  |  |  |  |
| **ASSESSMENTS：** |  |  |  |  |  |  |
| **Basic characteristics** | **×** |  |  |  |  |  |
| **FMA-UE** | **×** |  |  |  |  | **×** |
| **MAS** | **×** |  |  |  |  | **×** |
| **WMFT** | **×** |  |  |  |  | **×** |
| **SIS** | **×** |  |  |  |  | **×** |
| **Kinect** | **×** |  |  |  |  | **×** |
| **sEMG** | **×** |  |  |  |  | **×** |
| **EEG** | **×** |  |  |  |  | **×** |
| ***fNIRS*** | **×** |  |  |  |  | **×** |
| **Adverse events** |  |  | **×** | **×** | **×** | **×** |

**Fig. 2** SPIRIT figure. Schedule of enrolment, interventions, and assessments
