# Supplementary material for: Neurobiomechanical mechanism of Tai Chi to improve upper limb coordination function in post-stroke patients: a study protocol for a randomized controlled trial
Source: Trials. 2023 Dec 4;24:788. doi: 10.1186/s13063-023-07743-w (PMC10696787; doi:10.1186/s13063-023-07743-w)
Supplement: Supplementary file 5 — Additional file 5. Model consent form. [file 13063_2023_7743_MOESM5_ESM.docx]

**Consent Form**

**——Informed page**

Dear Volunteers,

We invite you to participate in the research of the neurobiomechanical mechanism by which Tai Chi improves the upper limb movement strategy of poststroke patients. This research is a doctoral project of the Fujian University of Traditional Chinese Medicine, and 84 volunteers are expected to participate. This study has been reviewed and approved by the Ethics Committee of the Rehabilitation Hospital Affiliated with the Fujian University of Traditional Chinese Medicine and conforms to relevant laws and the Declaration of Helsinki.

Please read the following content carefully to help you understand the purpose, significance, and method of the study, as well as the benefits, risks, or discomfort it may bring. You can discuss with your relatives and friends or ask researchers to explain to help you decide whether to participate.

Thank you for your support!

1. **Why should this research be conducted?**

Stroke is one of the most common causes of adult dysfunction, and rehabilitation intervention is urgently needed. The most severe limb damage after stroke is damage to the contralateral upper limb, including loss of motor ability, coordination, sense, and dexterity. Although many studies have been carried out to improve functional recovery, due to the limited understanding of the neurophysiological mechanism underlying motor recovery and the lack of adequate long-term interventions, upper limb motor recovery is still a challenging problem. The persistent inability to recover the upper limb will lead to disability and a decline in quality of life.

Tai Chi is a traditional Chinese Qigong exercise that focuses on the integration of body and mind and emphasizes the combination of mind, qi, and shape, which helps improve the coordination of upper limbs. Previous studies have confirmed that Tai Chi can improve the upper limb motor function of poststroke patients. However, the mechanism by which Tai Chi improves this function is still unclear, which has limited its clinical application.

Research shows that recovery of upper limb motor function in poststroke patients depends on functional reorganization at the cortical level and cortical muscle reorganization. Neurocoupling of sensorimotor network changes in poststroke patients, and the excitability of the corticospinal cord decreases. Tai Chi or exercise training can activate sensorimotor-related brain areas in ordinary people, and Tai Chi can improve the excitability of corticospinal pathways in poststroke patients. Therefore, we propose that Tai Chi regulates the reorganization of the ipsilateral sensorimotor cortex and cortical muscles in poststroke patients, thus improving their upper limb motor function.

This study aims to explore the effect of Tai Chi on the upper limb motor function of poststroke patients and its neural mechanism and to provide the theoretical basis for clinical application. We believe that this trial will have important theoretical and practical significance to improve upper limb function of poststroke patients.

**2. Who can participate in this study?**

This study intends to recruit 84 poststroke patients (subjects), mainly from Fujian Provincial Rehabilitation Hospital, who meet the following diagnosis and inclusion criteria, and those who meet the exclusion criteria should not participate in the study.

**Diagnostic criteria**

The diagnostic criteria of ‘stroke’ in the Diagnostic Essentials of Various Cerebrovascular Diseases adopted at the Fourth National Conference on Cerebrovascular Diseases in 1995 have been adopted.

**Inclusion criteria**

To be eligible to participate in the study, a participant must meet the following criteria:

1. Meet the diagnostic criteria of ‘stroke’ in the diagnostic essentials of various cerebrovascular diseases adopted at the Fourth National Cerebrovascular Disease Academic Conference in 1995 and confirmed by head computed tomography or magnetic resonance imaging;

2. The first stroke occurred between 2 weeks and 6 months prior to commencing the study;

3. Unilateral cortical lesion (left or right hemisphere) or subcortical lesion involving the motor pathway;

4. The muscle strength of the upper limb of the affected side is ≥ 3 and muscle tone is ≤ 2 based on the Modified Ashworth Scale (MAS);

5. Right-handedness before stroke;

6. Age between 50 and 70 years;

7. Blood pressure stable and below 160/100 mmHg;

8. A Mini-Mental State Examination (MMSE) score of > 17 points for illiterate participants, > 20 points for participants with a primary school education, and > 24 points for participants with a middle school and above education;

9. Brunnstrom stage ≥ IV in the affected upper limb;

10. Standing balance ≥ level 2, the ability to stand independently for more than 5 minutes, and the ability to walk independently for more than 6 metres;

11. Willing to sign the informed consent form and understand, accept, and implement the rehabilitation guidance.

**Exclusion criteria**

A participant who meets the following criteria will be excluded:

1. Upper limb motor dysfunction caused by other diseases such as brain tumours, brain injury, or parasitic brain disease;

2. Diseases affecting the ability to participate in Tai Chi training: serious lower limb joint diseases, arthritis, joint injury, cervical spondylotic myelopathy, lumbosacral spinal canal stenosis, and lower limb neuropathy;

3. Severe complications of stroke, such as severe pulmonary infection, shoulder hand syndrome, or venous embolism of lower limbs;

4. Severe heart disease; heart, liver, and kidney failure; malignant tumours; and gastrointestinal bleeding;

5. MMSE score ≤ 17 points for illiterate participants, ≤ 20 points for participants with a primary school education, and ≤ 24 points for participants with a middle school and above education;

6. Severe visual impairment and cannot complete the training;

7. Sensory aphasia (unable to understand the instructions);

8. Participation in Tai Chi training within the past 6 months;

9. Contraindications for near-infrared functional imaging and EEG, such as skin infection, scalp wound, dermatitis, and metal implants under electrodes;

10. Skin or muscle lesions of extremities that affect surface EMG;

11. Unable to cooperate or not suitable to participate in the examination, evaluation, and treatment of this study for other reasons, such as intolerable pain, abnormal mental state, or limited ability to move; and

12. Participation in other clinical studies.

**3. What do you need to be done if you participate in this study?**

1. Before you are enrolled in the study, the researchers will ask for and record your basic information and your health history, and they will assess you according to the trial criteria (diagnostic criteria, inclusion criteria, and exclusion criteria). If you meet the inclusion criteria of this study, you can voluntarily participate in the study and sign the informed consent form.
2. After signing the informed consent form, the researchers will carry out systematic baseline data recording, including basic information such as age and gender and examination and evaluation of motor function.
3. According to the principle of random distribution, you will have a 50% chance of being placed into one of two group:

a. Control group: during the test, the subjects receive routine rehabilitation training: five 60-minute sessions a week for a total of 4 weeks.

b. Tai Chi group: in addition to routine rehabilitation training, the subjects receive simplified Tai Chi training including eight poses under the guidance of a professional Tai Chi instructor. The training involves five 60- minute sessions a week for a total of 4 weeks.

Researchers will inspect and evaluate relevant indicators before and after the intervention for 4 weeks.

You can continue to use conventional drugs, but to ensure the rigor of the study, please do not regularly practice other aerobic exercises during the study. The investigator will record the medications you are taking at the baseline assessment. Please complete the Subject Activity Log issued by the researcher regularly every week to record your weekly medication and exercise. In addition, please strictly cooperate with the training. If you cannot complete the training within the specified time, you will be deemed to have automatically quit.

**4. Possible benefits and significance of participating in research**

As a participant in this study, you will receive all the examinations described in the study plan free of charge, including two unmarked induction kinematics assessments, near-infrared brain functional imaging examinations, electroencephalography, and surface electromyography. You will also receive free access to professional Tai Chi training, free rehabilitation guidance related to research content and free consultation related to stroke rehabilitation. After this study, you will receive a subsidy of 300 RMB.

Throughout this study, your condition may improve. This study will help to determine which treatment method can more safely and effectively treat and improve upper limb motor function in poststroke patients.

**5. Possible adverse reactions, risks, and discomfort in the study**

No dangerous events related to Tai Chi treatment have been reported. Possible adverse reactions during the study period could include dizziness, fatigue, or falls during or after the Tai Chi training. If the above adverse reactions occur during this study, the researcher should be informed in a timely manner. The researcher will make a judgment, give appropriate treatment, and record the event. The researcher will try to prevent discomfort and adverse reactions this study may cause. Possible adverse reactions and corresponding prevention and treatment measures are as follows:

(1) Subjects may experience muscle soreness and fatigue during or after Tai Chi. If the above adverse reactions occur, the instructor will decide whether to continue or immediately stop the test according to the severity and guide the patients to take necessary measures such as appropriate rest and warm compress to relieve.

(2) The instructor will do everything possible to prevent the subject from falling during training. The principles of falling prevention mainly include avoiding fatigue training, avoiding slippery and dark space training, avoiding crowded training, and controlling good training order. In the case of a fall, the training will be stopped immediately, and the injured or suspected injured person will be sent to the hospital.

**6. Other instructions**

Your personal information, assessment and treatment information, medical records, etc., will be kept by the research group, and researchers, research authorities, and the ethics committee will be allowed to access your medical records. Any public reports on the results of this study will not disclose your identity. We will make every effort to protect the privacy of your data within the scope permitted by law.

Participation in this study is entirely voluntary. You can refuse to participate in the study or withdraw from the study at any time, and participation will not affect the treatment you receive from your doctor. We will inform you in time if we get information that affects your ability to continue participating in the trial.

**7. Contact information**

(1) Contact information of researchers:

Xie Qiurong: 15005000559

(2) Contact information of the ethics committee

Medical Ethics Committee of Rehabilitation Hospital Affiliated to Fujian University of Traditional Chinese Medicine: 0591-88529126

**Consent Form**

**——Agree on the Signature Page**

Project name: Study on the neuro-biomechanical mechanism of Tai Chi to improve the upper limb movement strategy of poststroke patients

Undertaking unit: Fujian University of Traditional Chinese Medicine

Volunteer statement:

I have read the above introduction to this study and fully understand the risks and benefits that may arise from participating in this study. After full consideration, I have agreed to participate in this study and comply with the relevant work arrangements of the project subject.

I agree□ or refuse□ that studies other than this one use my research data.

Signature of volunteer:

Signature of authorized agent:

Relationship between the authorized agent and the subject:

Contact information of volunteer (authorized agent):

Date: Year Month Day

Statement of the researcher:

I confirm that I have explained the details of this study to the volunteers, especially the possible risks and benefits of participating in this study.

Signature of researcher:

Researcher's work phone:

Date: Year Month Day
